# Correction: Evaluation of in-service training program of laboratory professionals in Amhara Public Health Institute Dessie Branch, northeast Ethiopia: A concurrent mixed-method study

**DOI:** 10.1371/journal.pone.0249429

**Published:** 2021-03-25

**Authors:** Seid Legesse, Tefera Alemu, Mulugeta Tassew, Birtukan Shiferaw, Semagn Amare, Zerfie Tadesse, Minwuyelet Maru

The first two authors, Seid Legesse and Tefera Alemu, should be noted as contributing equally to this work.

The third, fourth, and fifth authors, Mulugeta Tassew, Birtukan Shiferaw, and Semagn Amare, should be noted as having also contributed equally to this work.

## References

[1] Legesse S, Alemu T, Tassew M, Shiferaw B, Amare S, Tadesse Z, et al. (2020) Evaluation of in-service training program of laboratory professionals in Amhara Public Health Institute Dessie Branch, northeast Ethiopia: A concurrent mixed-method study. PLoS ONE 15(12): e0243141. 10.1371/journal.pone.0243141 33275629 PMC7717548

