# Supplementary material for: Evaluation of in-service training program of laboratory professionals in Amhara Public Health Institute Dessie Branch, northeast Ethiopia: A concurrent mixed-method study
Source: PLoS One. 2020 Dec 4;15(12):e0243141. doi: 10.1371/journal.pone.0243141 (PMC7717548; doi:10.1371/journal.pone.0243141)
Supplement: S1 Appendix — (DOCX) [file pone.0243141.s001.docx]

# S1 Appendix: Training Evaluation Tool

**Part I: Quantitative Tool**

|  | **SECTION 1: SOCIODEMOGRAPHIC AND TYPE OF TRAINING TAKEN**  Name of Health facility; _____________________________ Date of Interview---------------  Type of health facility: 1. Hospital 2. Health center  Sex: 1. Male 2. female  Age in years --------------- Work experience in years -------------  Level of education ------------------ | | | | | | | | | | | | | | | | | | | |
| --- | --- | --- | --- | --- | --- | --- | --- | --- | --- | --- | --- | --- | --- | --- | --- | --- | --- | --- | --- | --- |
|  | Type of training /Training Course/ | | Trained | | | | | Training given by (Organization) | | | | | | | | | | | | |
|  |  |  | Yes | No | | | |  |  |  |  |  |  |  |  |  |  |  |  |  |
|  | HIV Rapid test | |  |  | | | |  | | | | | | | | | | | | |
|  | TB & FM /ZN Diagnosis | |  |  | | | |  | | | | | | | | | | | | |
|  | Malaria diagnosis | |  |  | | | |  | | | | | | | | | | | | |
|  | LQMS | |  |  | | | |  | | | | | | | | | | | | |
|  | SLMTA | |  |  | | | |  | | | | | | | | | | | | |
|  | Safety & Biosecurity | |  |  | | | |  | | | | | | | | | | | | |
|  | Automation (ART) | |  |  | | | |  | | | | | | | | | | | | |
|  | DBS/Viral load sample collection | |  |  | | | |  | | | | | | | | | | | | |
|  | Sample Referral linkage | |  |  | | | |  | | | | | | | | | | | | |
|  | Microbiology (Basic) | |  |  | | | |  | | | | | | | | | | | | |
|  | Gene x-pert |  | | |  | | |  | | | | | | | | | | | | |
|  | Others-specify |  | | |  | | |  | | | | | | | | | | | | |
|  | **SECTION 2: COURSE STRUCTURE AND CONTENT** | | | | | | | | | | | | | | | | | | | |
|  | **Interview items** | | | | | **Strongly**  **Disagree** | | | **Disagree** | | **Can Not**  **Decide** | | | | | **Agree** | | | **Strongly Agree** | |
| 1 | Training goals and objectives clearly stated before you started the training that you have taken? | | | | |  | | |  | |  | | | | |  | | |  | |
| 2 | The training length was sufficient to deliver the content that you have taken? . | | | | |  | | |  | |  | | | | |  | | |  | |
| 3 | The course design (i.e., materials and learning activities) is encouraging and enough for the training. | | | | |  | | |  | |  | | | | |  | | |  | |
| 4 | The quality of the content is consistent throughout the course in each training? | | | | |  | | |  | |  | | | | |  | | |  | |
| 5 | Was the content in-depth enough? | | | | |  | | |  | |  | | | | |  | | |  | |
| 6 | The course provided opportunities to practice and reinforce what was taught. | | | | |  | | |  | |  | | | | |  | | |  | |
| 7 | The course information was at an appropriate level to understand the learning objectives. | | | | |  | | |  | |  | | | | |  | | |  | |
| 8 | The course outcome was successful compared to your expectations? | | | | |  | | |  | |  | | | | |  | | |  | |
| 9 | The trainee selection criteria in your facility is based on responsibility | | | | |  | | |  | |  | | | | |  | | |  | |
|  | **SECTION 3: TRAINING TOOLS** | | | | | | | | | | | | | | | | | | | |
| 1 | The learning aids (i.e., workbooks, hand-outs, role-playing exercises, PowerPoint slides, software) assisted your learning. | | | | |  | | |  | |  | | | |  | |  | | | |
| 2 | The technology/lab equipment was working properly during the training. | | | | |  | | |  | |  | | | |  | |  | | | |
| 3 | There was more than one training style used that was conducive to my learning style (i.e. straight lecture, lecture with visual aids and/or interaction). | | | | |  | | |  | |  | | | |  | |  | | | |
|  | **SECTION 4: TRAINERS KNOWLEDGE AND SKILL VALUATION** | | | | | | | | | | | | | | | | | | | |
| 1 | Was The trainer prepared for class? | | | | | |  | | |  | |  | |  | | | |  | | |
| 2 | The trainer was knowledgeable about the course | | | | | |  | | |  | |  | |  | | | |  | | |
| 3 | How would you rate your trainer’s communication skills? | | | | | |  | | |  | |  | |  | | | |  | | |
| 4 | How would you rate your trainer’s delivery skills? | | | | | |  | | |  | |  | |  | | | |  | | |
| 5 | The trainer was responsive to questions and other needs. | | | | | |  | | |  | |  | |  | | | |  | | |
| 6 | The instructor encouraged a participatory and interactive learning environment. | | | | | |  | | |  | |  | |  | | | |  | | |
|  | **SECTION 5: NEW KNOWLEDGE AND SKILL ACQUISITION (LEARNING LEVEL)** | | | | | | | | | | | | | | | | | | | |
| 1 | Do you feel that your knowledge or skills have improved by taking the course? | | | | | |  | | |  | |  | |  | | | |  | | |
| 2 | Do you believe that the practical exercises were good that actually improved your skill | | | | | |  | | |  | |  | |  | | | |  | | |
| **SECTION 6: TRAINING SET UP & ENVIRONMENT EVALUATION** | | | | | | | | | | | | | | | | | | | |  |
| 1 | The training facilities/hall, toilet, café, etc/ were suitable for learning. | | | | | |  | | |  | |  | |  | | | |  | |  |
| 2 | There was adequate accommodation/perdium/tea break for the training | | | | | |  | | |  | |  | |  | | | |  | |  |
| 3 | The training location was easy to find | | | | | |  | | |  | |  | |  | | | |  | |  |
| 4 | The training season/given time was appropriate | | | | | |  | | |  | |  | |  | | | |  | |  |
| 5 | The number of trainee in one hall was not too large | | | | | |  | | |  | |  | |  | | | |  | |  |
| 6 | The training call was done in appropriate time | | | | | |  | | |  | |  | |  | | | |  | |  |
| 7 | The training call was heard in appropriate time | | | | | |  | | |  | |  | |  | | | |  | |  |
| 8 | Training call modality using direct phone call is appropriate for better communication | | | | | |  | | |  | |  | |  | | | |  | |  |
| 9 | Training call modality through e-mail to zone or facility head is appropriate for better communication | | | | | |  | | |  | |  | |  | | | |  | |  |
| **SECTION 7: CHANGE IN WORK BEHAVIOR AND PERFORMANCE** | | | | | | | | | | | | | | | | | | | |  |
|  | **Interview Items** | | | | | | | | | | | | **Yes** | | | | | **No** | |  |
| 1 | Were the learned knowledge and gained skills used in a workplace? | | | | | | | | | | | |  | | | | |  | |  |
| 2 | Do you have any suggestions that would make this course better? | | | | | | | | | | | |  | | | | |  | |  |
| 3 | Would you take a training like this again? | | | | | | | | | | | |  | | | | |  | |  |
| 4 | Would you recommend these trainings to a colleague? | | | | | | | | | | | |  | | | | |  | |  |
| 5 | Would you consider further training on the topic on your own? | | | | | | | | | | | |  | | | | |  | |  |
| 6 | Would you like to have received some further reading material | | | | | | | | | | | |  | | | | |  | |  |
| 7 | Identified thematic area for training | | | | | | | | | | | |  | | | | |  | |  |
| 8 | Do you have monitoring and evaluation system | | | | | | | | | | | |  | | | | |  | |  |
| 9 | Organization announce when trained personnel change their work site | | | | | | | | | | | |  | | | | |  | |  |
| 10 | Is the training materials on use/shared to other staffs in your work place | | | | | | | | | | | |  | | | | |  | |  |
| 11 | Trying to qualify other health professionals in your work place | | | | | | | | | | | |  | | | | |  | |  |
| 12 | Assign trained personnel’s on proper work position | | | | | | | | | | | |  | | | | |  | |  |
| 13 | Is there registration system that indicate trained staffs in the lab unit | | | | | | | | | | | |  | | | | |  | |  |

**Part 2. Qualitative guiding questions (Write the answers on the space provided)**

1. Can you tell us the major strengths and weakness on the course structures, training contents and training tools of APHI DB in service trainings?
2. Are there any comments/suggestions that you want to add on trainee’s reaction towards the course structures, training contents and training tools of APHI DB in service training?
3. How do you think we can improve/solve the weakness/limitations on the course structures, training contents and training tools of the training program?
4. What are the major strengths and weakness on trainer’s knowledge on the course and their communication skill during the training?
5. Do you have any other comments/suggestions that you want to add on trainer’s knowledge and their communication skill?
6. Can you tell us the way how we solve the weakness/limitation you mentioned on trainer’s knowledge on the course and their communication skill during the training?
7. Is there anything which has changed your knowledge, skill and attitude as a result of taking the training course? (New knowledge and skills acquired (Learning level)
8. Did you incorporate anything else you learned in the training course into your work? (Change in job behavior and performance (Behavioral level)
9. Can you tell us the major strengths and weakness on the training set up and environments of APHI DB in service training center?
10. Do you have any comments/suggestions that you want to add on the training set up and environments of APHI DB?
11. Anything else you what to add on APHI DB in service training program?
