# Supplementary material for: Evaluation of in-service training program of laboratory professionals in Amhara Public Health Institute Dessie Branch, northeast Ethiopia: A concurrent mixed-method study
Source: PLoS One. 2020 Dec 4;15(12):e0243141. doi: 10.1371/journal.pone.0243141 (PMC7717548; doi:10.1371/journal.pone.0243141)
Supplement: Attachment — Submitted filename: Review Comments_Ethopia Training evaluation paper.docx [file pone.0243141.s002.docx]

**Review Comments**

Paper Title: **Evaluating the effectiveness of in-service training program of Amhara Public Health Institute Dessie, Branch, Northeast Ethiopia: a concurrent nested mixed quantitative/qualitative based cross-sectional study**

1. The title is too long and does not reflect what was actually studied. An effectiveness study needs to have well allied outcome measures.
2. While I do understand that the authors may not be native English speakers, the paper is fill with grammatic errors and inconsistencies.
3. Your findings are not directly related to conclusions. Authors indicated that “…. only 65.6% of participants transferred their knowledge and skills in to practice”. This is a major outcome measure, but the authors failed to indicate how it was measured.
4. The introduction is inconsistent and difficult to follow. It starts with objectives, then to needs, gaps, and then back to objectives. This confuses the readers to know which of the objectives they are out address.
5. It is clear that the investigators collected the data themselves. What is not very clear is if they were the trainers? If they were, the results may be skewed because the trainees (in this case the respondents) will want to please the researchers especially in a qualitative design.
