# Supplementary material for: Evaluation of in-service training program of laboratory professionals in Amhara Public Health Institute Dessie Branch, northeast Ethiopia: A concurrent mixed-method study
Source: PLoS One. 2020 Dec 4;15(12):e0243141. doi: 10.1371/journal.pone.0243141 (PMC7717548; doi:10.1371/journal.pone.0243141)
Supplement: Attachment — Submitted filename: Response to Reviewers.docx [file pone.0243141.s003.docx]

Dear PLOS ONE

Her is authors point by point response letter for the comments raised by each of the reviewers/editors. The authors would like to thank the reviewers for their valuable comments. Care has been taken to improve the work and address their concerns as per the specific comments below.

Editor #1.

Comment 1: 1. The context of the training is not clear - are these standard training programs delivered by a national agency or varied programs delivered by a range of groups? The authors do mention the training institution but this could be described in more detail in the introduction to provide context. Further, the objectives and nature of training could be described - the results detail the topics and skills covered but it is not clear if the training is a single course designed to cover all these topics, a range of courses covering separate topics, or something else? Do participants select courses based on the topics covered/time of offering etc or are they directed to attend? Do participants attend more than one course? do workplaces support their employees to attend...etc etc. Please provide this context in the introduction.

Response 1: Comment accepted and incorporated. You can see under paragraph 2 and 3 of the introduction section.

Comment 2: 2. The introduction talks about both laboratory inservice and quality of care improvement training but it seems that the study relates to the former only - again, please clarify the context and objectives of this study and the training being evaluated.

Response 2: Comment accepted and modified. The objective of this study is to evaluate the effectiveness of in service training program of Amhara public health institute that delivers different capacity building trainings for laboratory professionals working under government health facilities. Therefore, this study did not evaluate a single training type; rather it has tried to evaluate the overall in service training program effectiveness based on predefined evaluation criteria that are common to most training types.

Comment 3: 3. More detail is needed to describe the survey and qualitative data collection methods. There is a reference to 'visits' - was data collected in person? How many people completed the survey, and how many participated in interviews?

Response 3: A total of 107 key informants/stakeholders were interviewed in the qualitative data collection. The qualitative questions are embedded in to the quantitative parts. The data is collected through face to face interviews. This is explained in the method section line number………

Comment 4: 4. The results could be presented more succinctly - the tables are unnecessarily detailed (e.g. you don't need the frequency for both yeas and no responses). Results could be more meaningful if they were linked to the objectives of the training, and you could also link the outcomes with participants' level of experience etc.

Response: 4: Comment accepted. Actually most of our tables responses are categorized based on likert scale which is mandatory to present all responses in a table. It is only table 2 and 6 that are classified as yes or no response. We believe that it seems a missing data if we omit either of yes or no response.

Comment 5: 4. Please provide more detail about the thematic analysis methods.

Response 5*:* Also the qualitative data was narrated and summarized based on four thematic areas, i.e. (i) Trainees reaction towards training, (ii) Trainers knowledge on the course & their communication skills, (iii) Learning of training content, (iii) Changes in job behavior after attending training (iv)Training set ups, benefits and financial issues. See under data analysis section.

Comment 6: 5. The discussion and conclusions could be improved by providing a more clear direction in terms of future directions and recommendations for improvement of training programs.

Response 6: Comment accepted. We revised based on comments. See the track changes under discussion section.

Comment 8: 1. Please ensure that your manuscript meets PLOS ONE's style requirements, including those for file naming.

Response 8: Comment accepted and revised according to the journal guideline.


Comment 9: 3. Please include additional information regarding the survey or questionnaire used in the study and ensure that you have provided sufficient details that others could replicate the analyses. For instance, if you developed a questionnaire as part of this study and it is not under a copyright more restrictive than CC-BY, please include a copy, in both the original language and English, as Supporting Information.

Response 9: Comment accepted and evaluation tool is uploaded as supporting information file.

Comment 10: 4. We note that you have provided funding information that is not currently declared in your Funding Statement. However, funding information should not appear in the Acknowledgments section or other areas of your manuscript. We will only publish funding information present in the Funding Statement section of the online submission form.

Please remove any funding-related text from the manuscript and let us know how you would like to update your Funding Statement.

Response 10: Comment accepted and funding related statement is deleted from the acknowledgement section.

 Comment 11: 6. Please amend your list of authors on the manuscript to ensure that each author is linked to an affiliation. Authors’ affiliations should reflect the institution where the work was done (if authors moved subsequently, you can also list the new affiliation stating “current affiliation:….” as necessary).

Response 11: Comment accepted and corrected.

Comment 12: 7. We note you have included tables to which you do not refer in the text of your manuscript. Please ensure that you refer to Tables 3. 4, 5, 6, 7 in your text; if accepted, production will need this reference to link the reader to the Tables.

 Response 13: Comment accepted and tables are cited in the manuscript.

Comment 14: 8. Please upload a copy of Supporting Information which you refer to in your text on page 15 (Availability of data and materials section).

[Response 14: Comment aaccepted. Data collection tool is uploaded as supporting information 1.

Reviewers' comments:

Reviewer #1

Comment 1: 1. The title of the paper is too long. Can you please consider a shorter but attention grapping title?

Response 1: Comment accepted and title is modified.

Comment 2: 2. I do understand that the authors may not be native English speakers, the paper is filled with grammatical errors and inconsistencies.

Response 2: Comment accepted. The manuscript is edited with someone fluent in English.
Comment 3; 3. Authors indicated that “…. only 65.6% of participants transferred their knowledge and skills in to practice”. This is a major outcome measure, but the authors failed to indicate how it was measured.

## Response 3; A total of 10 questions were used to measure trainees change in job behavior and performance in their workplace and the average is found to be 65.6%( see table 6). Comment 4: 4. The introduction is inconsistent and difficult to follow. It starts with objectives, then to needs, gaps, and then back to objectives. This confuses the readers to know which of the objectives the authors are out address.

Response 4; Comment accepted. We have tried to rewrite the introduction part to keep ideas coherent. You can see the track change version of the manuscript.
Comment 5: 5. It is clear that the investigators collected the data themselves. What is not very clear is if they were the trainers? If they were, the results may be skewed because the trainees (in this case the respondents) will want to please the researchers especially in a qualitative design.

Response 5: The investigators/data collectors are from research and technology transfer, public health emergency, monitoring and evaluation and human resource directorates, and none of them are from laboratory directorate. Therefore, none of them are mandated to provide trainings for laboratory professionals.

Comment 6. Authors should avoid blank statements that suggest a pre- and post-training assessments. This is evident in third paragraph of the discussion section.
Response 6: Comment accepted and modified accordingly. See paragraph three under discussion section.
