# Supplementary material for: Evaluation of in-service training program of laboratory professionals in Amhara Public Health Institute Dessie Branch, northeast Ethiopia: A concurrent mixed-method study
Source: PLoS One. 2020 Dec 4;15(12):e0243141. doi: 10.1371/journal.pone.0243141 (PMC7717548; doi:10.1371/journal.pone.0243141)
Supplement: Attachment — Submitted filename: Response to Reviewers 2.docx [file pone.0243141.s004.docx]

Dear PLOS ONE

Her is authors point by point response letter for the comments raised by the editor. The authors would like to thank the academic editor for his valuable comments. Care has been taken to improve the work and to address each specific comment. You can see the details of our modification under the track change version of the manuscript.

Additional Editor Comments (if provided)

Comment 1: Line 29 – suggest ‘involved interviews with each of these key stakeholders’

Response 1: Comment accepted and corrected. See under method section of the abstract and main manuscript.
Comment 2: Line 34 –The abstract needs some re-working for language. The results are presented as ‘x% agree/strongly agree with…’ doesn’t fit grammatically. I suggesting changing to “…agree/strongly agree that the trainers had adequate knowledge and skills….” Etc etc

Response 2: Comment accepted and corrected.
Comment 3: Line 50 – replace could with should

Response 3: accepted and word replaced.
Comment 4: Line 109 – here it says 110 respondents – then in line 113 it says 107 - what happened to 3 participants?/which number is correct?

Response 4: Comment accepted and modified. Actually 110 is the calculated sample size for the study and 107 is the actual number of study participants.
Comment 5: Line 113 – add ‘in-person’ and the location in which the interviews occurred

Response 5: Comment accepted and modified. See under data collection subsection.
Comment 6: Line 115 ‘close’ should be ‘closed’

Response 6: accepted and modified.
Comment 7: Lines 152 and 153 – I am unclear what you mean by diploma holders vs first degree professionals – can you please explain the qualifications of respondents?

Response 7: Comment accepted and modified. It is to mean laboratory technicians and BSc degree graduates in medical laboratory. See line number 154 under result section.
Comment 8: Line 164 – should be algorithm?

Response 8: It is to mean the new national HIV testing procedure. It is nationally agreed to be called “New National HIV Testing Algorism” training.
Comment 9: Table 2 – please just show the yes results. Being dichotomous, the ‘no’s’ can be assumed from this

Response 9: Comment accepted and the No response is deleted. See table 2 line number 172 and table 6 line number 226.
Comment 10: Line 205 – ‘as a potential to confident to the trainers’ – this needs to be re-worded

Response 10: Comment accepted and corrected.
Comment 11: Line 231 – “Agreeing with the training set-up” doesn’t make sense grammatically – was the question that it was appropriate/adequate/conducive to learning…please clarify

Response 11: Comment accepted and modified.
Comment 12: Table 8 talks about the ‘effectiveness’ of different elements – I suggest using ‘effective’ where describing the learning and transfer of knowledge to the workplace, but ‘appropriate’ or ‘adequate’ may be better for other elements such as the training set up

Response 12: Comment accepted and the words are modified. See table 8 line number 250

Comment 13: I was also interested in the comment about the judgment regarding access to perdiem – I think this needs some exploration as it is unclear why this would occur and in what context.
Response 13: Comment accepted and corrected. See under discussion section line number 291 and 292.
