# Supplementary material for: Evaluation of in-service training program of laboratory professionals in Amhara Public Health Institute Dessie Branch, northeast Ethiopia: A concurrent mixed-method study
Source: PLoS One. 2020 Dec 4;15(12):e0243141. doi: 10.1371/journal.pone.0243141 (PMC7717548; doi:10.1371/journal.pone.0243141)
Supplement: Attachment — Submitted filename: Response to Reviewers 3.docx [file pone.0243141.s005.docx]

Dear PLOS ONE

Her is authors response letter for the comments raised by the editor. You can see the details of our modification under the track change version of the manuscript.

Additional Editor Comments (if provided):

Comment 1: The authors have done a comprehensive job of addressing the previous comments but there remain problematic language and grammatical errors in the paper. I request that you pursue professional copyediting to enable further consideration of this manuscript.

Response 1: Comment accepted and corrected. The manuscript is edited with someone else who is fluent in English language. Besides, as you can see the track change version of the manuscript, care has been taken to improve the work and to address language problems by the authors.
